# An internally and externally validated nomogram for predicting cancer-specific survival in octogenarians after radical resection for colorectal cancer

**DOI:** 10.1007/s40520-024-02809-4

**Published:** 2024-07-26

**Authors:** Junchang Zhu, Wei Cen, Xuzhi Zheng, Chenqiao Ye, Feifan Guo, Xialin Yan, Hongying Shi, Lechi Ye, Tingting Hu

**Affiliations:** 1 Department of Colorectal and Anal Surgery, First Affiliated Hospital of Wenzhou Medical University, Wenzhou, China; 2 Department of Epidemiology and Health Statistics, School of Public Health and Management, Wenzhou Medical University, Wenzhou, China; 3 Department of Gastroenterology and Hepatology, First Affiliated Hospital of Wenzhou Medical University, Wenzhou, China

**Keywords:** Nomogram, Octogenarian, Colorectal cancer

## Abstract

**Aims:**

We aimed to develop an elaborative nomogram that predicts cancer-specific survival (CSS) in American and Chinese octogenarians treated with radical resection for CRC.

**Methods:**

The patient data of newly diagnosed patients aged 80 years or older who underwent radical resection for CRC from 2010 to 2015 were extracted from the Surveillance, Epidemiology, and End Results (SEER) database and then randomly divided into a training cohort and a validation cohort. The patients collected from our hospital were defined as the external validation cohort. Univariate and multivariate Cox regression was used to select independent predictive factors for the construction of a nomogram to predict 1-, 2- and 3-year CSS.

**Results:**

The multivariate Cox regression model identified age, T stage, N stage, perineural invasion, chemotherapy, tumour deposits, carcinoembryonic antigen level, number of lymph node metastases, and number of solid organ metastases as independent predictors of survival. The C-index of the nomogram for 1-, 2- and 3-year CSS was 0.758, 0.762, and 0.727, respectively, demonstrating significant clinical value and substantial reliability compared to the TNM stage. The calibration curve and area under the curve also indicated considerable predictive accuracy. In addition, decision curve analysis demonstrated desirable net benefits in clinical application.

**Conclusion:**

We constructed a nomogram for predicting the CSS of individual octogenarian patients with CRC who underwent radical resection. The nomogram performed better than the TNM staging system in this particular population and could guide clinicians in clinical follow-up and individual therapeutic plan formulation.

**Supplementary Information:**

The online version contains supplementary material available at 10.1007/s40520-024-02809-4.

## Highlights

This study explored the exclusive characteristics of octogenarians treated with radical resection for colorectal cancer.

This study is the first to develop a nomogram for predicting the cancer-specific survival of individual octogenarian patients after radical resection for colorectal cancer with the substantial reliability (C-index: 0.758 compared to the AJCC pathological staging system (C-index: 0.713).

Internal validation of this nomogram in a cohort of American patients (*N* = 1644) and external validation in a cohort of Chinese patients (*N* = 368) confirmed its accuracy.

**Supplementary Information:**

The online version contains supplementary material available at 10.1007/s40520-024-02809-4.

## Introduction

As the population ages, the proportion of octogenarians (patients over 80 years old) is rapidly increasing [1–3]. Colorectal cancer (CRC) is the third most frequent cancer diagnosed among men and women in the United States, posing an age-specific risk [4]. According to the latest statistics, the incidence rate escalates rapidly with age, with 121.4 per 100,000 population in individuals aged 60 to 64 years compared with 237.9 per 100,000 aged 80 to 84 [5]. Thus, octogenarians are becoming an essential cohort of CRC patients.

Octogenarians have been historically recognized as a population at higher risk for undergoing major surgical procedures due to the severity of their comorbidities and decreased functionality. This perspective has been modified recently, and age is no longer considered a contraindication to abdominal surgery [6–8]. However, among researchers, controversy persists regarding the prognosis of patients in this cohort. Several studies have shown poorer survival after surgery in older CRC patients than in younger patients [9–12], although some studies have concluded that the survival of older patients is quite good [6, 13, 14]. Thus, a pragmatic and customized approach is required to evaluate the prognostic factors and prognosis of octogenarians treated with CRC resection.

Nomograms are currently the most precise method for predicting the prognosis of cancer patients [15]. Although Zhang et al. [16]. constructed postoperative nomograms to predict cancer-specific survival (CSS) after CRC resection, Wang et al. [17]. also developed a predictive model of cause-specific death (CSD) in elderly patients (age ≥ 65 years) using a competing-risk approach. However, a cut-off of 65 years is not a fair representation of the older population based on demographic ageing [18, 19]. None of these previous studies have targeted the emerging clinical population of octogenarians [7]. Meanwhile, the present method of estimating the prognosis of patients in this particular population is based on the American Joint Committee on Cancer (AJCC) TNM stage. At the same time, the TNM stage does not include independent prognostic factors such as tumour deposits (TDs) [20], perineural invasion (PNI) [20], or carcinoembryonic antigen (CEA) level [21], which tends to result in reduced accuracy.

Therefore, we compiled information on octogenarian patients after radical resection for CRC from the National Cancer Institute’s Surveillance, Epidemiology, and End Results (SEER) database and from the Colorectal Surgery Department of the First Affiliated Hospital of Wenzhou Medical University. We developed a nomogram to analyse and investigate the prognostic factors associated with CSS in this particular population and predicted their CSS to guide individualized treatment planning.

## Method

### Patient population and selection

**Fig. 1:**
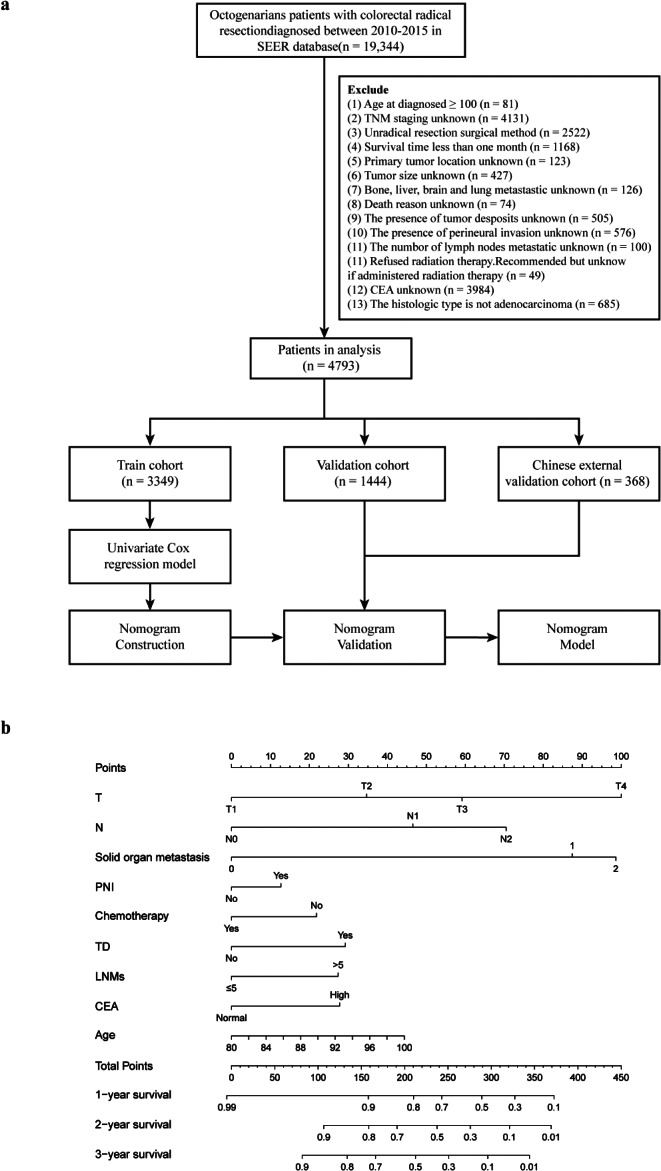
**a** Flow chart for the study of octogenarians treated with radical resection for colorectal cancer according to the inclusion and exclusion criteria, with internal validation in an American cohort and external validation in a Chinese cohort. **b** Nomogram prediction of 1-, 2- and 3-year CSS in octogenarians in the training cohort after radical resection for CRC. The nomogram is applied using points scored according to each involved variable, as indicated on the point scale. The total point score represents the probability of CSS at various points in time, with a higher score signifying a higher potential for an undesirable outcome

The SEER program represents the principal source of a national database and cancer statistics in America and is presently maintained by the National Cancer Institute (NCI) [22]. The patient data of newly diagnosed CRC patients aged 80 years or older from 2010 to 2015 were extracted from the SEER database using the SEER*Stat program (v 8.4.0.1) [22]. Demographic information, clinicopathological information, treatment information, and follow-up results were collected. Deaths from cancer were characterized by SEER CSD classification variables. The initial inclusion criteria were as follows: (1) age ≥ 80 years; (2) pathological diagnosis of CRC and histologic type of adenocarcinoma; (3) year of diagnosis from 2010 to 2015; and (4) complete survival dates. The exclusion criteria were as follows: (1) histologic type other than adenocarcinoma; (2) survival ≤ 1 month; and (3) incomplete clinical information (data for most patients older than 100 years are incomplete). A total of 19,344 patients were collected and screened for inclusion in the study (*n* = 4794) (Fig. 1a). Then, the included individuals were randomly divided into a training cohort (70%) and a validation cohort (30%). The training cohort was used to construct the nomogram, while the smaller validation cohort was used to validate the model.

External validation data were finally derived from 368 octogenarian patients who underwent radical resection for CRC in the Department of Colorectal and Anal Surgery of the First Affiliated Hospital of Wenzhou Medical University from January 2016 to January 2020. The time of the last follow-up was January 2023. The inclusion and exclusion criteria were as described above. The research was approved by the ethics committees of the hospital and conformed to the seventh version of the Declaration of Helsinki.

### Variable collection

Several variables were included in the current study: baseline demographics (i.e., age at diagnosis, sex); tumour features (i.e., T stage, N stage, tumour size, histologic type, tumour location, TDs, PNI, number of metastatic lymph nodes (LNs), CEA level, number of solid organ (liver, lung, brain, bone)) metastases; treatment information (i.e., chemotherapy, radiation therapy); and survival variables (i.e., survival duration in months, vital status). We restaged all octogenarians according to the eighth edition of the AJCC pathological staging system [23]. The tumour size was stratified by a 5 cm cut-off. In our study, CSS was determined as the duration from diagnosis to death or last contact and was used as the primary outcome.

### Statistical method and nomogram construction

Categorical variables in the analysis are described as percentages, and continuous variables are described as the median and interquartile range (IQR). Comparisons of categorical covariates were performed via the chi-square test or Kruskal–Wallis test, and comparisons of continuous covariates were performed by the Mann‒Whitney U test. Then, univariate Cox regression was applied to filter the variables that were significantly associated with CSS in the training group. Predictors with p values less than 0.05 were entered into a multivariate Cox regression model. Variables were further eliminated using backwards stepwise selection based on the Bayesian information criterion (BIC) [24]. The obtained multivariate Cox regression models were used to evaluate the hazard ratios (HRs) and construct the ultimate prognostic nomogram model for predicting CSS at 1, 2, and 3 years in octogenarians after radical resection for CRC.

### Nomogram validation

The functionality of the nomogram was determined by authentication and calibration utilizing both the internal and external validation cohorts. The concordance index (C-index) was adopted to evaluate the accuracy and discriminatory ability of the nomogram. The method “Bootstrap” described by Harrel is employed to ascertain the statistically significant value of the C-index between different models [25]. The calibration curve and area under the receiver operating characteristic (ROC) curve (AUC) were used to assess the calibration. Meanwhile, the Delong’s test is employed to compare the AUC values of different models. Nomogram validation was performed utilizing an internal validation set and an independent external validation set satisfying the previously indicated inclusion criteria.

### Clinical utility

Decision curve analysis (DCA) is an approach to estimate the utility of a model by determining the net benefit under different thresholds [26, 27]. We assessed the clinical applicability of the predictive nomogram model by DCA and compared it to the AJCC TNM staging system. Meanwhile, the patients were divided into high-risk and low-risk groups based on the ROC cut-off values, and the variation in the survival of patients in each risk group was detected using the log-rank test and Kaplan‒Meier (K‒M) curve. All statistical analyses were conducted using SPSS 22.0 (SPSS, Chicago, IL, USA) and R software 4.2.1 (http://www.r-project.org). A two-sided test was statistically validated at *P* < 0.05.

## Results

### Patient characteristics

A total of 5,162 patients were recruited for this research; among them, 3349 were in the training cohort, 1445 were in the validation cohort, and 368 were in the external validation cohort (Fig. 1a). The population characteristics and clinicopathological features of the training and validation cohorts (internal and external) are shown in Table 1. There were no statistically significant differences in baseline data between the training and internal validation data sets (*P* > 0.05).

**Table 1:** Clinicopathological characteristics of octogenarians after curative resection

Variables	Training cohort^†^ (*N* = 3349)	Validation cohort^†^ (*N* = 1444)	*P*	External cohort (*N* = 368)
Age (years)	84(82,87)	84(82,88)	0.389	83(81,85)
Sex			0.391	
Male	1423(42.5%)	633(43.8%)		162(44.0%)
Female	1926(57.5%)	811(56.2%)		206(56.0%)
Stage			0.639	
I	677(20.2%)	297(20.6%)		45(12.2%)
II	1382(41.3%)	562(38.9%)		122(33.2%)
III	1071(32.0%)	507(35.1%)		144(39.1%)
IV	219(6.5%)	78(5.4%)		57(15.5%)
T			0.321	
T1	226(6.7%)	107(6.8%)		27(7.3%)
T2	562(16.8%)	245(17.0%)		106(28.8%)
T3	2031(60.6%)	878(60.8%)		195(53.0%)
T4	530(15.8%)	214(14.8%)		40(10.9%)
N			0.119	
N0	2119(63.3%)	881(61.0%)		186(50.5%)
N1	809(24.2%)	363(25.1%)		145(39.4%)
N2	421(12.6%)	200(13.9%)		37(10.1%)
Tumour size (cm)			0.533	
≤ 5 cm	2384(62.2%)	1001(60.9%)		157(76.2%)
>5 cm	1450(37.8%)	643(39.1%)		49(23.8%)
Tumour location			0.544	
Colon	3111(92.9%)	1334(92.4%)		336(91.3%)
Rectum	238(7.1%)	110(7.6%)		32(8.7%)
Number of solid organ (liver, lung, brain, bone) metastases			0.191	
No metastasis	3130(93.5%)	1366(94.6%)		312(84.8%)
One solid organ metastasis	193(5.8%)	65(4.5%)		45(12.2%)
More than one solid organ metastasis	26(0.8%)	13(0.9%)		11(3.0%)
Tumour desposits			0.476	
Yes	366(10.9%)	147(10.2%)		59(16.0%)
No	2983(89.1%)	1297(89.8%)		309(84.0%)
Perineural invasion			0.192	
Yes	325(9.7%)	158(10.9%)		259(70.4%)
No	3024(90.3%)	1286(89.1%)		109(29.6%)
Number of metastatic lymph nodes			0.717	
≤ 5	3108(92.8%)	1336(92.5%)		351(95.4%)
>5	241(7.2%)	108(7.5%)		17(4.6%)
Chemotherapy			0.498	
Yes	546(16.3%)	247(17.1%)		70(19.0%)
No/Unknown	2803(83.7%)	1197(82.9%)		298(81.0%)
Radiation therapy			0.293	
Yes	139(4.2%)	50(3.5%)		3(0.8%)
No/Unknown	3210(95.8%)	1394(96.5%)		365(99.2%)
CEA level			0.525	
Normal	1905(56.9%)	836(57.9%)		206(56.0%)
High	1444(43.1%)	608(42.1%)		162(44.0%)

### Nomogram construction

The univariate analysis showed that age, sex, tumour size, number of solid organ metastases, TDs, PNI, number of metastatic LNs, chemotherapy, and CEA level were statistically significantly related to CSS (Table 2**)**. Following stepwise selection to eliminate further potential redundancies, the resulting nomogram model included age, T stage, N stage, number of solid organ metastases (liver, lung, brain, bone), TDs, PNI, number of metastatic LNs, chemotherapy and CEA level (Table 2**)**. The risk scores for the individual items were calculated by summing the scores using the nomogram (Fig. 1b).

**Table 2:** Cox regression of univariate and multivariate analyses associated with CSS in octogenarians after curative resection

Variables	Univariate analysis			Multivariate analysis		
	HR	95% CI	*P* value	HR (95%)	95% CI	*P* value
Age (years)	1.032	1.015–1.048	< 0.001^***^	1.033	1.016–1.050	< 0.001^***^
Female	0.875	0.774–0.990	0.035^*^	0.940	0.829–1.066	0.332
T						
T1	Ref			Ref		
T2	1.776	1.104–2.835	0.018^*^	1.633	1.015–2.629	0.043^*^
T3	3.630	2.349–5.610	< 0.001^***^	2.296	1.475–3.575	< 0.001^***^
T4	10.075	6.473–15.682	< 0.001^***^	4.103	2.597–6.482	< 0.001^***^
N						
N0	Ref			Ref		
N1	2.657	2.299–3.071	< 0.001^***^	1.952	1.663–2.290	< 0.001^***^
N2	5.882	5.042–6.861	< 0.001^***^	2.748	2.172–3.476	< 0.001^***^
Tumour size (>5 cm)	1.297	1.144–1.470	< 0.001^***^	1.039	0.914–1.182	0.556
Tumour location (rectum)	0.966	0.763–1.224	0.776			
Number of solid organ (liver, lung, brain, bone) metastases						
No metastasis	Ref					
One solid organ metastasis	6.668	5.625–7.904	< 0.001^***^	3.417	2.824–4.135	< 0.001^***^
More than one solid organ metastasis	8.488	5.594–12.878	< 0.001^***^	4.011	2.620–6.142	0.001^***^
Tumour desposits	3.634	3.137–4.210	< 0.001^***^	1.499	1.273–1.766	< 0.001^***^
Perineural invasion	2.602	2.210–3.063	< 0.001^***^	1.207	1.014–1.437	0.035^*^
Number of metastatic lymph nodes (> 5)	4.660	3.949–5.499	< 0.001^***^	1.471	1.157–1.871	0.002^**^
Chemotherapy	1.933	1.682–2.222	< 0.001^***^	0.738	0.629–0.867	< 0.001^***^
Radiation therapy	1.189	0.902–1.568	0.219			
CEA level (high)	2.062	1.822–2.335	< 0.001^***^	1.488	1.308–1.693	< 0.001

* Show statistically significant difference s P

### Nomogram both internal and external validation

This research examined the model’s reliability through cross-validation with internal cohorts and independent validation with external cohorts of Chinese patients. First, we validated the accuracy of the nomogram with the C-index. In the training, validation and external validation cohorts, the C-index values of the nomogram (training cohort, 0.758 (95% CI: 0.742–0.774); internal validation cohort, 0.762 (95% CI: 0.738–0.787)) were higher than those based on the AJCC stage (training cohort, 0.713 (95% CI: 0.697–0.729, *p* = 0.002); internal validation cohort, 0.701 (95% CI: 0.677–0.725, *p* = 0.003)), demonstrating the good discriminatory ability of the model.

**Fig. 2:**
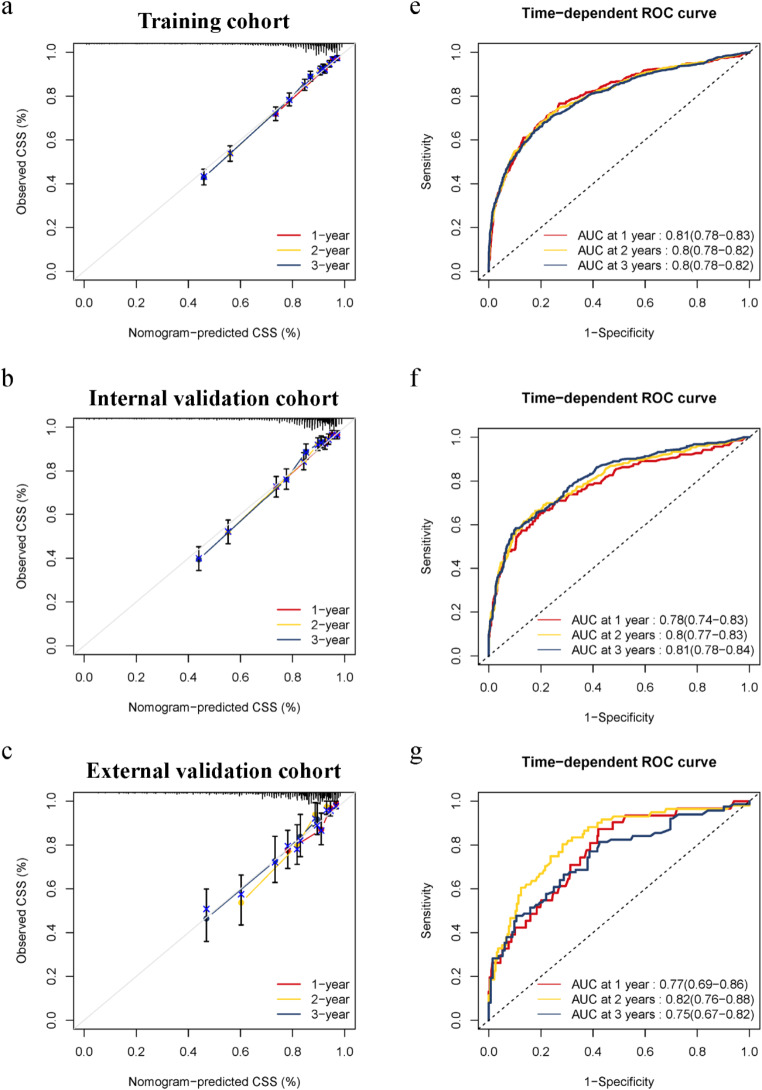
Calibration of the nomogram in the training and validation cohorts and the AUC in the training and validation cohorts. **a** The 1-, 2-, and 3-year CSS in the training cohort. **b** The 1-, 2-, and 3-year CSS in the internal validation cohort. **c** The 1-, 2-, and 3-year CSS in the Chinese external cohort. **e-g** The AUC in the training, internal and external cohorts

This model was simultaneously validated in an external validation group. The C-index values of the model (external validation cohort, 0.727 (95% CI: 0.677–0.777)) were higher than AJCC stage (external validation cohort, 0.644 (95% CI: 0.586–0.702, *p* = 0.005)), which further validated the model’s reliability. The calibration curves for the training and validation cohorts revealed that the forecasts of the predictive model corresponded to the actual observed values (Fig. 2a-i).

The AUC in these three cohorts also indicated comparable efficiency of the model in distinguishing the effect. In the training cohort, the AUC of 1-, 2- and 3-year were 0.81 (95% CI: 0.78–0.83)), 0.8 (95% CI: 0.78–0.82)) and 0.8 (95% CI: 0.78–0.82)), respectively (Fig. 2e). In the internal validation cohort, the AUC of 1-, 2- and 3-year were respectively 0.78 (95% CI: 0.74–0.83)), 0.8 (95% CI: 0.77–0.83)) and 0.81 (95% CI: 0.78–0.84)) (Fig. 2f), while in the external validation cohort, the AUC of 1-,2- and 3-year were 0.77 (95% CI: 0.69–0.86)), 0.82 (95% CI: 0.76–0.88)) and 0.75 (95% CI: 0.67–0.82). Compared to the model of AJCC TNM, the nomogram also exhibits the greater predictive ability (Fig.S1 a-c).

### Clinical application of the nomogram

**Fig. 3:**
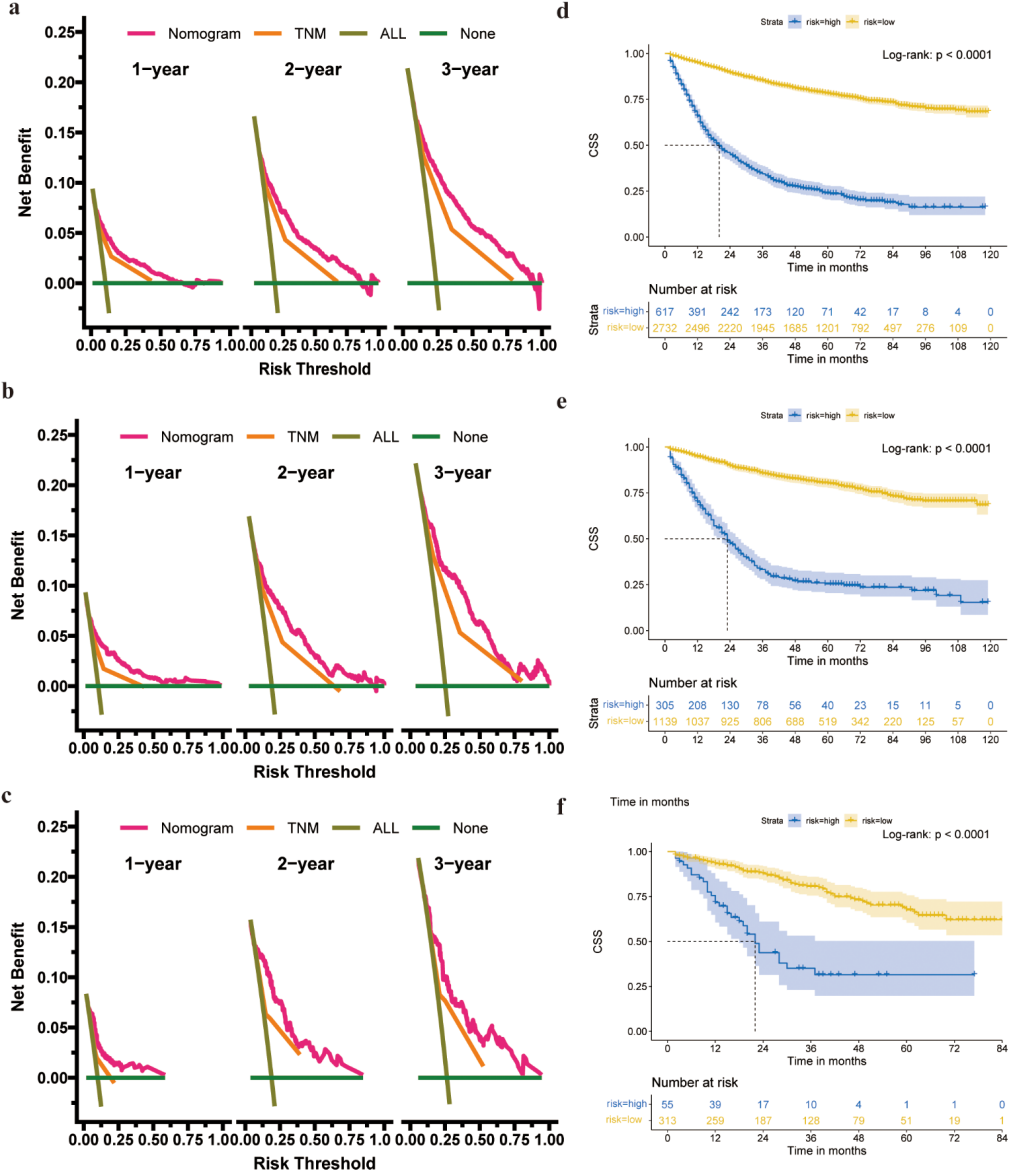
DCA and KM results in the training and validation cohorts. The X-axis represents the probability of death, while the Y-axis indicates the net benefit of the therapy for patients. The dark green colour represents the net benefit with the risk threshold when all patients survive. The horizontal light green line indicates the net benefit of therapy when all patients die. The red line represents the nomogram, and the yellow line represents the TNM stage. The clinical value of the nomogram is better than that of the TNM stage when the risk threshold is between 0 and 100%. **a-c** DCA results in the training, internal, and external validation cohorts. **d**-f The KM results of training cohort (**d**), internal validation cohort (**e**) and external validation cohort(**f**)

DCA illustrated higher clinical value for the nomogram than the TNM staging system in the training, validation and external validation cohorts (Fig. 3a-c). The patients were then separated into a high-risk group (total score ≥ 159.5) and a low-risk group (total score < 159.5) based on the cut-off value of the ROC curve. The 1-year, 2-year, and 3-year survival rate in the high-risk group was 65.8%, 45.1%, and 34.3%, respectively. In the low-risk group, the 1-year, 2-year, and 3-year survival rate was 94.9%, 89.5%, and 85.2%, respectively. Based on K–M curves and log-rank tests, the survival rates in the high-and low-risk groups in these three cohorts were remarkably distinct, with significantly lower survival rates in high-risk patients than in low-risk patients (Fig. 3d-f).

## Discussion

The necessity of exploring the characteristics of older populations, such as octogenarians, is increasing with increasing human life expectancy [18, 19]. However, research in this particular population is considerably restricted for many reasons, especially research on CRC. First, the high incidence of frailty and preoperative comorbidities or postoperative complications in octogenarians predisposes them to make fewer visits. Thus, controversy persists regarding the prognosis and improvement in the quality of life of octogenarians after surgery because of the limited data for this population [7, 28, 29]. Thus, the demographic information of elderly patients with CRC and their corresponding pathological features have commonly been investigated with a cut-off of 65 years of age [16, 17, 30]. Studies on octogenarians have generally focused on the impact of postoperative complications on their survival [12, 28]. However, recent studies have indicated that perioperative complications have no impact on the short-term survival of octogenarians [9, 13]. This leads to the exclusive characteristics of this population being easily neglected.

In this study, we developed a prognostic nomogram model using the American SEER database and validated its accuracy by internal cross-validation. In addition, our study considered the distinctive features of the Chinese and American patient populations. We externally validated the model with a Chinese single-centre database (*N* = 368) to exclude time and geographical influences, demonstrating the model’s precision. Compared by these two cohorts, we found that China has a higher rate of solid organ metastasis than the United States due to inadequate CRC surveillance, which resulted in an imbalance among the three groups at baseline [31]. In addition, chemotherapy and radiotherapy were not widely used in these three groups. One reason for this may be that the benefits of chemotherapy and radiotherapy have not been demonstrated in older adults over 70 years of age; another reason may be that older adults have poorer physical function and often poorly adapt to chemotherapy and radiotherapy [32].

In this nomogram, age, T stage, N stage, PNI, chemotherapy, TDs, CEA level, number of metastatic LNs, and number of solid organ metastases were chosen after univariate Cox regression screening and backwards stepwise selection. From the results, we constructed a model in the training set with potential benefits in predicting CSS in octogenarians after radical resection for CRC based on demographic information and clinicopathological traits.

Age at diagnosis was recognized as an independent prognostic factor in our nomogram. Although the exact mechanism has not been elucidated, similar conclusions have been proposed by numerous studies [13, 17, 33]. Similarly, the patterns of LNM in CRC are continually clarified, and the recognition of LNM is associated with decreased survival in CRC patients [4, 5, 34]. Considering the complicated physical characteristics of octogenarians, we performed modelling using continuous variables in pursuit of the most accurate predictions. It is traditionally perceived that chemotherapy in octogenarians represents a tremendous challenge to physical function and may lead to a worse outcome. However, our study found a survival advantage in octogenarians receiving chemotherapy, which mirrors the conclusions of some studies [35–37]. In addition, we innovatively incorporated TDs and PNI into our model, both of which have been proven to be substantially associated with the prognosis of CRC patients [20]. There is increasing evidence that TDs are associated with LNM, extramural vascular invasion (EMVI) and PNI, which are markers of a poorer prognosis, with potential additive effects of the combination of these features [38–40]. Restricted by the patient information in the SEER database, we predicted the prognosis of CRC patients using all three.

The AJCC TNM staging system presents many limitations regarding octogenarians. According to the TNM staging system, octogenarians with disease of similar stages may present with similar prognoses, which does not meet the practical requirements. Compared to the TNM staging system, our nomogram provided a more precise analysis of this sensitive group, with a C-index of 0.758, good calibration, and distinctive DCA results. Thus, the nomogram established in this study could be used to guide clinicians in individual therapeutic plan formulation and patient follow-up. For example, in the high-risk group (total score ≥ 159.5), necessary follow-ups are performed at certain times to confirm the postoperative status and enable treatment plan adjustment. In the low-risk group (total score < 159.5), a more aggressive therapeutic intervention might be chosen, depending on the patient’s wishes and the experimental conditions, to prolong the life expectancy and improve the quality of life of octogenarians. Additionally, our nomogram was less predictive of CSS in China. The primary reason might be that China faced a wave of Omicron infections at the end of 2022. Octogenarians infected with Omicron faced a greater challenge to their physical function, resulting in poorer CSS. Another reason might be that the tumour metastasis rate in octogenarians is higher in China than in the United States, resulting in a poorer prognosis. This may also reflect the inadequate system for early CRC screening in China [41, 42] and highlight the need to predict CRC in this population more accurately.

The prognostic nomogram developed in this study has significant clinical and practical implications for the management of colorectal cancer (CRC) in octogenarians. Octogenarians, distinguished by a shorter life expectancy and a higher prevalence of morbidity, frequently encounter challenges in reconciling therapeutic approaches with their desired quality of life. Meanwhile, the ongoing updating of theoretical framework and the relentless advancement of technology have made it challenging for geriatricians to balance the potential benefits and inherent dangers of aggressive treatments for octogenarians. The nomogram represents a valuable tool for personalized risk assessment, as it enables the identification of high-risk patients who may benefit from closer monitoring and tailored treatment strategies. Conversely, low-risk patients may be managed with a standard follow-up regimen, thus helping to set realistic expectations and make informed decisions about patient care. Concurrently, the nomogram facilitates geriatricians in conveying complex prognostic information to patients and their families in a straightforward and comprehensible pictorial format, thereby enhancing patient comprehension, satisfaction, and adherence to treatment plans. Furthermore, in complex clinical settings, the nomogram assists geriatricians in identifying patients who necessitate more intensive resources and those who can be managed with routine care, thereby optimizing the allocation of healthcare resources.

There are also some limitations to our study. First, the SEER database is a high-quality population-based cancer registry lacking data on body mass index, smoking, underlying diseases, comorbidities, and other factors that may influence CSS in octogenarians. Second, selection bias may also be inevitable due to the retrospective study design. For instance, partially censored data associated with a short follow-up time can also statistically affect the performance of the predictive model. Third, our nomogram was validated with external data from a single centre in China. Thus, further validation of the nomogram using a large amount of data from multiple centres is needed.

## Conclusion

We constructed a nomogram for predicting the CSS of individual octogenarian patients with CRC who underwent radical resection. Internal validation in an American cohort and external validation in a Chinese cohort demonstrated the precision of the model and the better prognostic performance of the model than the conventional AJCC staging system in this particular population. Thus, this nomogram could be used to guide clinicians in clinical follow-up and therapeutic plan formulation.

### Electronic supplementary material

Below is the link to the electronic supplementary material.

Supplementary Material 1

Supplementary Material 2

Supplementary Material 3

## Data Availability

The data used to support the findings of this study are included within the article. The authors thank all patients and institutions involved in this study and are especially grateful for the ability to have open access to the SEER database.

## References

[1] European Commission B Population Structure and Ageing. [(accessed on 19 December 2022)]; Available online: https://ec.europa.eu/eurostat/statistics-explained/index.php?title=Population_structure_and_ageing

[2] Olshansky SJ, Goldman DP, Zheng Y, Rowe JW (2009) Aging in America in the twenty-first century: demographic forecasts from the MacArthur Foundation Research Network on an Aging Society. Milbank Q 87:842–862. 10.1111/j.1468-0009.2009.00581.x 20021588 10.1111/j.1468-0009.2009.00581.x PMC2888016

[3] Cancer Registry and Statistics (Monitoring of Cancer Incidence in Japan (MCIJ)). Cancer Information Service, National Cancer Center, Japan. https://ganjoho.jp/reg_stat/statistics/dl/index.html. Accessed 19 December 2022. In Japanese

[4] Siegel RL, Miller KD, Fuchs HE, Jemal A (2022) Cancer statistics, 2022. Cancer J Clin 72. 10.3322/caac.21708 10.3322/caac.21708 35020204

[5] Siegel RL, Miller KD, Goding Sauer A et al (2020) Colorectal cancer statistics, 2020. CA: a Cancer. J Clin 70:145–164. 10.3322/caac.21601 10.3322/caac.21601 32133645

[6] Pirrera B, Vaccari S, Cuicchi D et al (2016) Impact of octogenarians on surgical outcome in colorectal cancer. Int J Surg (London England) 35:28–33. 10.1016/j.ijsu.2016.09.006 10.1016/j.ijsu.2016.09.006 27616059

[7] Goldvaser H, Katz Shroitman N, Ben-Aharon I et al (2017) Octogenarian patients with colorectal cancer: characterizing an emerging clinical entity. World J Gastroenterol 23:1387–1396. 10.3748/wjg.v23.i8.1387 28293085 10.3748/wjg.v23.i8.1387 PMC5330823

[8] Neuwirth MG, Bierema C, Sinnamon AJ et al Trends in major upper abdominal surgery for cancer in octogenarians: has there been a change in patient selection? Cancer 2018; 124: 125–135. 10.1002/cncr.30977 10.1002/cncr.30977 28881379

[9] Duraes LC, Stocchi L, Dietz D et al (2016) The disproportionate effect of Perioperative complications on Mortality within 1 year after Colorectal Cancer Resection in octogenarians. Ann Surg Oncol 23:4293–4301 27459985 10.1245/s10434-016-5445-3

[10] Mothes H, Bauschke A, Schuele S et al (2017) Surgery for colorectal cancer in elderly patients: how can we improve outcome? J Cancer Res Clin Oncol 143:1879–1889. 10.1007/s00432-017-2438-y 28534171 10.1007/s00432-017-2438-y PMC11819352

[11] Pirrera B, Lucchi A, Gabbianelli C et al (2017) E.R.A.S. pathway in colorectal surgery in elderly: our experience: a retrospective cohort study. Int J Surg (London England) 43:101–106. 10.1016/j.ijsu.2017.05.013 10.1016/j.ijsu.2017.05.013 28483663

[12] Weerink LBM, Gant CM, van Leeuwen BL et al (2018) Long-term survival in octogenarians after Surgical Treatment for Colorectal Cancer: Prevention of Postoperative complications is key. Ann Surg Oncol 25:3874–3882. 10.1245/s10434-018-6766-1 30244418 10.1245/s10434-018-6766-1 PMC6245105

[13] Chan DKH, Leong SW, Keh CHL (2021) Perioperative and oncologic outcomes in young and octogenarian patients with colorectal cancer: a comparison at the extremes. Langenbeck’s Archives Surg 406:2399–2408. 10.1007/s00423-021-02275-w 10.1007/s00423-021-02275-w 34312720

[14] Schiffmann L, Ozcan S, Schwarz F et al (2008) Colorectal cancer in the elderly: surgical treatment and long-term survival. Int J Colorectal Dis 23:601–610. 10.1007/s00384-008-0457-5 18343931 10.1007/s00384-008-0457-5

[15] Shariat SF, Karakiewicz PI, Suardi N, Kattan MW (2008) Comparison of nomograms with other methods for predicting outcomes in prostate cancer: a critical analysis of the literature. Clin Cancer Research: Official J Am Association Cancer Res 14:4400–4407. 10.1158/1078-0432.CCR-07-4713 10.1158/1078-0432.CCR-07-4713 18628454

[16] Zhang Z-Y, Luo Q-F, Yin X-W et al (2016) Nomograms to predict survival after colorectal cancer resection without preoperative therapy. BMC Cancer 16:658. 10.1186/s12885-016-2684-4 27553083 10.1186/s12885-016-2684-4 PMC4995691

[17] Wang Z, Wang Y, Yang Y et al (2020) A competing-risk nomogram to predict cause-specific death in elderly patients with colorectal cancer after surgery (especially for colon cancer). World J Surg Oncol 18:30. 10.1186/s12957-020-1805-3 32019568 10.1186/s12957-020-1805-3 PMC7001222

[18] Christensen K, Doblhammer G, Rau R, Vaupel JW (2009) Ageing populations: the challenges ahead. Lancet (London England) 374:1196–1208. 10.1016/S0140-6736(09)61460-4 19801098 10.1016/S0140-6736(09)61460-4 PMC2810516

[19] Foreman KJ, Marquez N, Dolgert A et al (2018) Forecasting life expectancy, years of life lost, and all-cause and cause-specific mortality for 250 causes of death: reference and alternative scenarios for 2016-40 for 195 countries and territories. Lancet (London England) 392:2052–2090. 10.1016/S0140-6736(18)31694-5 30340847 10.1016/S0140-6736(18)31694-5 PMC6227505

[20] Mayo E, Llanos AAM, Yi X et al (2016) Prognostic value of tumour deposit and perineural invasion status in colorectal cancer patients: a SEER-based population study. Histopathology 69:230–238. 10.1111/his.12936 26802566 10.1111/his.12936

[21] Primrose JN, Perera R, Gray A et al (2014) Effect of 3 to 5 years of scheduled CEA and CT follow-up to detect recurrence of colorectal cancer: the FACS randomized clinical trial. JAMA 311:263–270. 10.1001/jama.2013.285718 24430319 10.1001/jama.2013.285718

[22] Surveillance E, Results E (SEER) Program (www.seer.cancer.gov) SEER*Stat Database: Incidence - SEER Research Plus Data, 12 Registries, Nov 2021 Sub (1992–2019) - Linked To County Attributes - Time Dependent (1990–2019) Income/Rurality, 1969–2020 Counties, National Cancer Institute, DCCPS, Surveillance Research Program, released April 2022, based on the November 2021 submission

[23] Amin MB, Greene FL, Edge SB et al (2017) The Eighth Edition AJCC Cancer staging Manual: continuing to build a bridge from a population-based to a more personalized approach to cancer staging. Cancer J Clin 67:93–99. 10.3322/caac.21388 10.3322/caac.21388 28094848

[24] Burnham KP, Anderson DR (2004) Multimodel inference: understanding AIC and BIC in model selection. Sociol Methods Res 33:261–304. 10.1177/0049124104268644 10.1177/0049124104268644

[25] Harrell FE, Lee KL, Mark DB (1996) Multivariable prognostic models: issues in developing models, evaluating assumptions and adequacy, and measuring and reducing errors. Stat Med 15:361–387 8668867 10.1002/(SICI)1097-0258(19960229)15:4<361::AID-SIM168>3.0.CO;2-4

[26] Van Calster B, Wynants L, Verbeek JFM et al (2018) Reporting and interpreting decision curve analysis: a guide for investigators. Eur Urol 74:796–804. 10.1016/j.eururo.2018.08.038 30241973 10.1016/j.eururo.2018.08.038 PMC6261531

[27] Tsalatsanis A, Hozo I, Vickers A, Djulbegovic B (2010) A regret theory approach to decision curve analysis: a novel method for eliciting decision makers’ preferences and decision-making. BMC Med Inf Decis Mak 10:51. 10.1186/1472-6947-10-51 10.1186/1472-6947-10-51 PMC2954854 20846413

[28] Høydahl Ø, Edna T-H, Xanthoulis A et al (2022) Octogenarian patients with colon cancer - postoperative morbidity and mortality are the major challenges. BMC Cancer 22:302. 10.1186/s12885-022-09384-9 35313841 10.1186/s12885-022-09384-9 PMC8939202

[29] Kunitake H, Zingmond DS, Ryoo J, Ko CY (2010) Caring for octogenarian and nonagenarian patients with colorectal cancer: what should our standards and expectations be? Dis Colon Rectum 53:735–743. 10.1007/DCR.0b013e3181cdd658 20389207 10.1007/DCR.0b013e3181cdd658

[30] Chen L-J, Nguyen TNM, Chang-Claude J et al (2022) Incorporation of functional status, frailty, comorbidities and comedication in prediction models for colorectal cancer survival. Int J Cancer 151:539–552. 10.1002/ijc.34036 35435251 10.1002/ijc.34036

[31] Ju W, Zheng R, Zhang S et al (2023) Cancer statistics in Chinese older people, 2022: current burden, time trends, and comparisons with the US, Japan, and the Republic of Korea. Sci China Life Sci 66:1079–1091. 10.1007/s11427-022-2218-x 36543994 10.1007/s11427-022-2218-x

[32] Benson AB, Venook AP, Al-Hawary MM et al (2022) Rectal Cancer, Version 2.2022, NCCN Clinical Practice guidelines in Oncology. J Natl Compr Cancer Network: JNCCN 20:1139–1167. 10.6004/jnccn.2022.0051 36240850 10.6004/jnccn.2022.0051

[33] Ge H, Yan Y, Xie M et al (2019) Construction of a nomogram to predict overall survival for patients with M1 stage of colorectal cancer: a retrospective cohort study. Int J Surg (London England) 72. 10.1016/j.ijsu.2019.10.021 10.1016/j.ijsu.2019.10.021 31678689

[34] Zhang C, Zhang L, Xu T et al (2020) Mapping the spreading routes of lymphatic metastases in human colorectal cancer. Nat Commun 11:1993. 10.1038/s41467-020-15886-6 32332722 10.1038/s41467-020-15886-6 PMC7181746

[35] Lai P, Sud S, Zhang T et al (2016) Palliative chemotherapy in advanced colorectal cancer patients 80 years of age and older. Curr Oncol (Toronto Ont) 23:144–153. 10.3747/co.23.2996 10.3747/co.23.2996 PMC4900825 27330342

[36] Zhang D, Wang X, Zhang M et al (2022) Clinical efficacy of chemotherapy in colorectal cancer patients over 80 years old. Int J Colorectal Dis 37:1853–1863. 10.1007/s00384-022-04222-7 35857106 10.1007/s00384-022-04222-7 PMC9388411

[37] Bergquist JR, Thiels CA, Spindler BA et al (2016) Benefit of postresection adjuvant chemotherapy for stage III Colon Cancer in octogenarians: analysis of the National Cancer Database. Dis Colon Rectum 59:1142–1149. 10.1097/DCR.0000000000000699 27824699 10.1097/DCR.0000000000000699

[38] Lord A, Brown G, Abulafi M et al (2021) Histopathological diagnosis of tumour deposits in colorectal cancer: a Delphi consensus study. Histopathology 79:168–175. 10.1111/his.14344 33511676 10.1111/his.14344

[39] Nagtegaal ID, Knijn N, Hugen N et al (2017) Tumor deposits in Colorectal Cancer: improving the Value of Modern Staging-A Systematic Review and Meta-analysis. J Clin Oncology: Official J Am Soc Clin Oncol 35:1119–1127. 10.1200/JCO.2016.68.9091 10.1200/JCO.2016.68.9091 28029327

[40] Delattre J-F, Selcen Oguz Erdogan A, Cohen R et al (2022) A comprehensive overview of tumour deposits in colorectal cancer: towards a next TNM classification. Cancer Treat Rev 103:102325. 10.1016/j.ctrv.2021.102325 34954486 10.1016/j.ctrv.2021.102325

[41] Zeng H, Ran X, An L et al (2021) Disparities in stage at diagnosis for five common cancers in China: a multicentre, hospital-based, observational study. Lancet Public Health 6:e877–e887. 10.1016/S2468-2667(21)00157-2 34838194 10.1016/S2468-2667(21)00157-2

[42] Chen H, Li N, Ren J et al (2019) Participation and yield of a population-based colorectal cancer screening programme in China. Gut 68:1450–1457. 10.1136/gutjnl-2018-317124 30377193 10.1136/gutjnl-2018-317124

