# Supplementary material for: An internally and externally validated nomogram for predicting cancer-specific survival in octogenarians after radical resection for colorectal cancer
Source: Aging Clin Exp Res. 2024 Jul 26;36(1):152. doi: 10.1007/s40520-024-02809-4 (PMC11282125; doi:10.1007/s40520-024-02809-4)
Supplement: Supplementary file 2 — Supplementary Material 2 [file 40520_2024_2809_MOESM2_ESM.pdf]

Editing Certificate

This document certifies that the manuscript

An internally and externally validated nomogram for predicting cancer-specific survival in octogenarians with colorectal cancer radical resection

prepared by the authors

Junchang Zhu;Wei Cen; Xuzhi Zheng;Chenqiao Ye;Lechi Ye;Tingting Hu

was edited for proper English language, grammar, punctuation, spelling, and overall style by one or more of the highly qualified native English speaking editors at AJE.

This certificate was issued on **March 8, 2023** and may be verified on the AJE website using the verification code **2D1D-19E8-7D73-4023-EA6F** .

Neither the research content nor the authors' intentions were altered in any way during the editing process. Documents receiving this certification should be English-ready for publication; however, the author has the ability to accept or reject our suggestions and changes. To verify the final AJE edited version, please visit our verification page at aje.com/certificate. If you have any questions or concerns about this edited document, please contact AJE at support@aje.com.

AJE provides a range of editing, translation, and manuscript services for researchers and publishers around the world.
For more information about our company, services, and partner discounts, please visit aje.com.
